# Are Smaller Emergency Departments More Prone to Volume Variability?

**DOI:** 10.5811/westjem.2021.2.49749

**Published:** 2021-07-14

**Authors:** Sara Nourazari, Jonathan W. Harding, Samuel R. Davis, Ori Litvak, Stephen J. Traub, Leon D. Sanchez

**Affiliations:** * California State University, Long Beach, Department of Health Care Administration, Long Beach, California; † Harvard Medical School, Beth Israel Deaconess Medical Center, Department of Emergency Medicine, Boston, Massachusetts; ‡ LogixHealth, Inc., Bedford, Massachusetts; § Brown University Warren Alpert Medical School, Department of Emergency Medicine, Providence, Rhode Island

## Abstract

**Introduction:**

Daily patient volume in emergency departments (ED) varies considerably between days and sites. Although studies have attempted to define “high-volume” days, no standard definition exists. Furthermore, it is not clear whether the frequency of high-volume days, by any definition, is related to the size of an ED. We aimed to determine the correlation between ED size and the frequency of high-volume days for various volume thresholds, and to develop a measure to identify high-volume days.

**Methods:**

We queried retrospective patient arrival data including 1,682,374 patient visits from 32 EDs in 12 states between July 1, 2018–June 30, 2019 and developed linear regression models to determine the correlation between ED size and volume variability. In addition, we performed a regression analysis and applied the Pearson correlation test to investigate the significance of median daily volumes with respect to the percent of days that crossed four volume thresholds ranging from 5–20% (in 5% increments) greater than each site’s median daily volume.

**Results:**

We found a strong negative correlation between ED median daily volume and volume variability (R^2^ = 81.0%; *P* < 0.0001). In addition, the four regression models for the percent of days exceeding specified thresholds greater than their daily median volumes had R^2^ values of 49.4%, 61.2%, 70.0%, and 71.8%, respectively, all with *P* < 0.0001.

**Conclusion:**

We sought to determine whether smaller EDs experience high-volume days more frequently than larger EDs. We found that high-volume days, when defined as days with a count of arrivals at or above certain median-based thresholds, are significantly more likely to occur in lower-volume EDs than in higher-volume EDs. To the extent that EDs allocate resources and plan to staff based on median volumes, these results suggest that smaller EDs are more likely to experience unpredictable, volume-based staffing challenges and operational costs. Given the lack of a standard measure to define a high-volume day in an ED, we recommend 10% above the median daily volume as a metric, for its relevance, generalizability across a broad range of EDs, and computational simplicity.

## INTRODUCTION

### Background

Emergency department (ED) visits in the United States increased from 119.2 million in 2006 to 145.6 million in 2016.^1^ The increase in visits contributes to crowding, boarding, and overtaxing of clinical staff capabilities.^2^,^3^ Several studies highlight the negative effects of crowding on patient satisfaction, care, health outcomes, and staff safety.^2^,^4^,^5^ Volume predictions and management strategies have been developed to improve operations and mitigate the impact of increased volume.^6^,^7^ Staffing all days to the level of high-volume days would reduce crowding, however, it would be costly and inefficient on lower-volume days. Staffing to the average demand is a common approach to balance these tradeoffs.

### Importance

A significant limitation of staffing to the average demand is that the method does not consider the day-to-day natural variability of demand, which is inherent to the system and cannot be eliminated. Although research exists on resource mobilization in a mass casualty or surge events (eg, the COVID-19 pandemic), few studies investigate the variability in patient volume on a day-to-day basis in the ED.^8^–^10^ A study demonstrating that lower-volume EDs are more prone to variability is of great value for effective and efficient management of ED operations and staffing. Furthermore, developing a measure for identifying high-volume days in EDs encourages robust staffing approaches, which could balance quality and efficiency while accounting for day-to-day volume variability.

### Goals

We compared the variability of patient volume relative to ED size by assessing volume-based thresholds (5%, 10%, 15%, and 20% greater than the daily median volume of the ED). We intentionally avoided standard deviations and percentiles, which naturally scale with ED volume. Using median-based thresholds as the standard measures, we studied whether smaller EDs experience a greater frequency of high-volume days as opposed to those of larger, more resource-heavy EDs.

## METHODS

### Data

This was a retrospective, observational study of aggregated third-party ED data. The dataset included 1,682,374 unique visits from 32 EDs in 12 states from July 1, 2018–June 30, 2019. The hospitals consisted of 28 urban and 4 rural hospitals. Collectively 5 out of 32 EDs were in academic hospitals, while the remaining 27 EDs were in community hospitals. We queried historical de-identified and anonymized data from a database of patient billing records provided by a national coding, billing, and analytics company (LogixHealth, Inc., Bedford, MA). The timestamps of patient arrivals were recorded and saved to a hospital database at the time of registration.

### Setting

We excluded from the analysis pediatric-only and freestanding EDs, as well as EDs lacking data for all 365 days. Median daily arrivals in the remaining EDs ranged from 79 to 214 resulting in the annual visits ranging from about 29,000 to about 78,000. It is worth noting that although this range is relatively broad, it may not be completely inclusive of extreme ED sizes.

### Analysis

To examine the correlation between ED median daily volume and volume variability, we developed a linear regression model with the following hypothesis:

H_0_: ED median daily volume and the variability of volume are not correlated.
H_1_: ED median daily volume and the variability of volume are linearly correlated.

Next, for all EDs we calculated the percent of days above 5%, 10%, 15%, and 20% of the median daily volume. We propose that smaller EDs will more frequently experience days with volume above a given threshold, defined as a percentage above their median daily volume. The structured hypothesis is as follows:

H_0_: The frequency of days that ED volume equals or exceeds 5%, 10%, 15%, and 20% of the median daily volume has no relation to the median daily volume of the ED.
H_1_: The frequency of days that ED volume equals or exceeds 5%, 10%, 15%, and 20% of the median daily volume is higher in EDs with a smaller median daily volume than those with a larger median daily volume.

We normalized the data to remove the day-of-week (DOW) effect. For each site, the ratio of the mean volume to the mean volume by DOW was multiplied by the true volume to generate adjusted daily volumes.

## RESULTS

**Figure 1:**
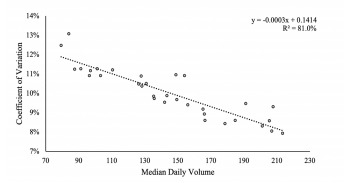
Regression analysis for coefficient of variation and median daily volume by emergency department: The coefficient of variation, which is equal to the standard deviation divided by the mean, is the dependent variable, while median daily volume for EDs is the independent variable. The results indicate a strong negative linear correlation with R^2^ of 81.0%.

To examine the correlation between volume variability (the dependent variable) and ED median daily volume (the independent variable), we calculated the coefficient of variation (COV) for each site. The COV is used to adjust variability for ED size. We then conducted a regression analysis to investigate the correlation between ED size and volume variability. The linear regression model follows the form of Y = mX+b, and here, X is a vector of the median daily volume for each of the EDs (the independent variable), while Y is a vector of the COV for each of the EDs (the dependent variable). The results displayed in Figure 1 indicate a strong negative correlation with R^2^ of 81.0% and *P* < 0.0001. These results demonstrate that smaller EDs generally have a higher COV and hence experience more daily volume variability than larger EDs.

**Figure 2:**
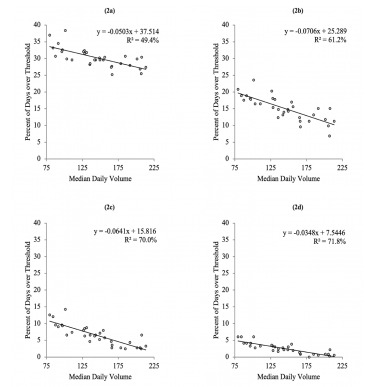
Regression analysis results: The percent of days exceeding specified thresholds vs daily median volume (2a: 5% above median volume, 2b: 10% above median volume, 2c: 15% above median volume, 2d: 20% above median volume). The data in all four charts indicate a negative slope, demonstrating that smaller emergency departments (ED) tend to cross percent-of-median volume thresholds more frequently than larger EDs. In these models, multiplying EDs median daily volume by the slope and adding the intercept produces an estimate of the percent of days that exceed the respective threshold.

We then developed a series of linear regression models and Pearson correlation tests (Figure 2) to test the primary study hypothesis. For these models, X is a vector of the median daily volumes for each of the EDs (the independent variable), while Y is a vector of the frequency of days equaling or exceeding a given threshold for each of the EDs (the dependent variable).

The results of the regression analysis indicate a statistically significant negative correlation between the independent and dependent variables, which led us to reject the null hypothesis for all four cases. This demonstrates that lower-volume EDs tend to experience high-volume days more frequently than higher-volume EDs. For instance, as shown in Figure 2c, the smaller EDs have days with 15% more volume than their median volume roughly four times as often as the larger EDs.

With the aim of formulating a measure to classify high-volume days that balances generalizability to various ED sizes, relevance, and derivation simplicity, we further analyzed the linear regression model results. To be able to generalize the high-volume metric to a broad range of EDs, we assessed the correlation determinations (R^2^) for which Figures 2b–d demonstrate sufficient quality.

Regarding the relevance of the metric, Figure 1a demonstrates that high-volume days with the threshold set to 5% above the median would occur about 25%–35% of the time, which is too common to be relevant for operational purposes. Figure 2b demonstrates that smaller EDs cross the 10% threshold on roughly 20% of days, whereas larger EDs cross the threshold on roughly 10% of days. Figures 2c and 2d illustrate that larger EDs almost never cross the 15% and 20% thresholds, which would prevent measures with these thresholds to be generalizable to a variety of EDs.

Given the overall regression quality, applicability to both large and small EDs, and simplicity of derivation, we recommend 10% above median daily volume to represent a reasonable threshold for identifying high-volume days in EDs. This proposed measure is the first step in developing comprehensive measures beyond the “average” or “median” daily volume to identify “busy” days in an ED and better capture a comprehensive view of daily volume variability.

## DISCUSSION

Although EDs vary with respect to the particulars of staffing, volume, acuity, boarding, and admission rate, they all are likely to operate differently on a low-volume day compared to a high-volume day. Unlike low-volume days, where different systems that are critical to efficient ED operation and flow are less likely to be stressed, higher volume days often lead to boarding and potential concerns for quality and safety because they strain medical resources and hinder the timeliness of emergency care. However, it is worth noting that low-volume days could also be problematic and impose financial challenges on ED operations as overstaffed days could lead to waste of resources and excess capacity. Hence, smaller EDs must develop strategies to identify, assess, and accommodate the effect and frequency of daily volume variability.

While the identified root causes of ED crowding and long wait times are predominantly linked to the inherent variability of demand, many of the existing solutions are focused on streamlining patient flow.^10^ Therefore, static solutions are being applied to a dynamic and unpredictable problem. Bridging this gap warrants the development and implementation of novel ED staffing approaches that adaptively align ED resources with demand. With the ability to classify high-volume days, ED leaders will be better equipped to proactively manage this variability and use appropriate staffing strategies that prevent prolonged wait times while balancing quality, provider satisfaction, operational complexity, and cost.

## LIMITATIONS

A limitation of this study is that some EDs naturally have more day-to-day variability than others. For instance, an ED in a seasonal vacation town may experience significantly higher volume in certain months. Future work could explore the benefit of including additional explanatory variables, such as specific ED location, to correct for this effect. Furthermore, we obtained the data in this study for EDs in only 12 states. Although these states were distributed across broad regions of the United States, further research is recommended to support generalizing the findings.

## CONCLUSION

Smaller EDs, in addition to having fewer resources to buffer increased demand, have more frequent high-volume days than larger EDs. Given the lack of a standard measure to define a high-volume day in EDs, we propose 10% above the median daily volume. Our recommended metric is directly related to daily ED volume and could be a starting point in identifying, understanding, and managing high-volume days in EDs. This work is a call to action for further studies in constructing a roadmap to develop robust measures that would help acknowledge, assess, and effectively plan for the daily volume variability in EDs.
